# *Entamoeba histolytica* Trophozoites Interact with the c-Met Receptor at the Surface of Liver Origin Cells through the Gal/GalNAc Amoebic Lectin

**DOI:** 10.3390/life11090923

**Published:** 2021-09-06

**Authors:** Jesus Pérez-Hernández, Clarisa Retana-González, Espiridión Ramos-Martínez, José Cruz-Colín, Andrés Saralegui-Amaro, Gabriela Baltazar-Rosario, Concepción Gutiérrez-Ruíz, Gerardo Aristi-Urista, Rosario López-Vancell

**Affiliations:** 1 Experimental Pathology Laboratory, Research Unit in Experimental Medicine, School of Medicine, National Autonomous University of Mexico, Mexico City 04519, Mexico; jespertex@yahoo.com.mx (J.P.-H.); clarisa.retana@gmail.com (C.R.-G.); espiri77mx@yahoo.com (E.R.-M.); gabriela.b.r@hotmail.com (G.B.-R.); 2 National Institute of Genomic Medicine, Mexico City 14610, Mexico; jcruz@inmegen.gob.mx; 3 National Laboratory for Advanced Microscopy, Institute of Biotechnology, National Autonomous University of Mexico, Cuernavaca, Morelos 62210, Mexico; sandres@ibt.unam.mx; 4 Cellular Physiology Laboratory, Biological and Health Sciences Division, Metropolitan Autonomous University, Mexico City 09340, Mexico; mcgr@xanum.uam.mx; 5 Pathology Service, General Hospital of Mexico “Dr. Eduardo Liceaga”, School of Medicine, UNAM (National Autonomous University of Mexico), Mexico City 06720, Mexico; gerardo.aristi.u@gmail.com

**Keywords:** Gal/GalNAc lectin, *Entamoeba histolytica* cytotoxicity, c-Met receptor

## Abstract

Amoebiasis in humans is caused by the protozoan parasite *Entamoeba histolytica,* which cytotoxic activity has been demonstrated on a wide variety of target cells. The process involves the adherence of the parasite to the cell, and such adherence is mediated by an amoebic surface lectin, known as Gal/GalNAc lectin. It is composed of heavy, intermediate, and light subunits. The carbohydrate recognition domain (CRD) has been identified within a cysteine-rich region in the lectin heavy subunit and has an amino acid sequence identity to the receptor-binding domain of hepatocyte growth factor (HGF). Recombinant CRD has been previously shown to compete with HGF for binding to the c-Met receptor IgG fusion protein. In the present study, we searched for evidence of interaction between the Gal/GalNAc lectin at the surface of trophozoites with the c-Met receptor expressed at the surface of HepG2 in coculture assays. Immunoprecipitation of the coculture lysate indicated interaction of the c-Met with a 60 kDa peptide recognized by antiamoebic lectin antibody. Colocalization of both molecules was detected by fluorescence confocal microscopy. Incubation of HepG2 cells with HGF before coculture with trophozoites prevents the cytotoxic effect caused by the parasites but not their adherence to the cells. Our results point to Gal/GalNAc lectin as a ligand of the c-Met receptor at the surface of HepG2 cells.

## 1. Introduction

Amoebiasis in humans is caused by *Entamoeba histolytica* whose cytotoxic activity on a variety of target cells has been widely demonstrated [1]. The process is contact dependent and involves adherence of the parasite through its surface lectin, known as Gal/GalNAc lectin (Gal lectin), which is composed of a 260 KDa heterodimer of disulfide-linked heavy (170 KDa) and light (35/31 KDa) subunits, which is noncovalently associated with an intermediate subunit of 150 KDa. The carbohydrate recognition domain (CRD) is localized in a cysteine-rich region in the heavy subunit from amino acids 895–998 [2]. The precise cytotoxic mechanism is unknown, but after adherence, host intracellular calcium becomes dramatically elevated, and host proteins become dephosphorylated contributing both events to cell death [3]. Although there has not been identified a specific receptor to which the Gal lectin binds, there are in the literature some reports related to pattern recognition receptors (PRRs). Chadee et al. [4] demonstrated that the amoebic lectin induced transcription of the Toll-like receptor 2 (TLR-2) gene in a murine macrophages cell line and that in TLR-2 gene regulation a mitogen-activated protein kinase (MAPK or MAP kinase) was involved; however, in that study, binding of the Gal lectin to the TLR-2 was not demonstrated. A later study showed that the recombinant CRD of the Gal lectin binds to TLR-2 and TLR-4 in human colonic cells and activates the classic signaling pathway of these receptors, concluding that the CRD of the amoebic Gal lectin worked similar to a pathogen-associated molecular pattern (PAMP) inducing expression of TLRs and inflammatory cytokines by binding to TLR receptors in the colonic cells [5].

When analyzing the CRD’s sequence, Dodson et al. [2] found there was some limited identity in sequence to the receptor-binding domain of hepatocyte growth factor (HGF). Specifically, the region from amino acids 913–939 of CRD had 52% sequence identity with amino acids 59–85 of HGF, which forms part of the receptor-binding domain sufficient for high-affinity HGF binding. The similarity was such that the recombinant CRD and even the purified Gal lectin competed with HGF for binding to the c-Met receptor in competition binding assays. The competition was not due to the carbohydrate-binding activity since the presence of *N*-acetylgalactosamine (GalNAc) in the assay did not modify the result. The biological consequences of this structural trait of Gal lectin have not yet been studied in in vitro cell coculture models. In the present study, we analyzed the interaction of trophozoites from *E. histolytica* HM-1:IMSS with HepG2 cells (a cell line derived from human hepatocarcinoma) through the amoebic Gal lectin (in the trophozoites’ membrane) and the c-Met receptor (at the HepG2 cells’ surface). The results obtained by immunoprecipitation with anti-c-Met antibodies after coculture revealed a band with an approximate molecular weight of 60 KDa recognized by anti-Gal lectin antibodies. We also found that both molecules, i.e., Gal lectin and c-Met, colocalized in cocultures of trophozoites and HepG2 cells, as shown by confocal fluorescence microscopy images. Finally, our results from a parallel microscopic study indicated that the cytotoxic effect caused by trophozoites to HepG2 cells was prevented by pretreatment of HepG2 cells with HGF before coculture with amoebic trophozoites, but the adherence of the trophozoites was still observed; the latter seems to involve the c-Met receptor in the cytotoxic effect.

## 2. Materials and Methods

### 2.1. Cell Culture

*Entamoeba histolytica* trophozoites, HM-1:IMSS, were axenically cultured in TYI-S-33 medium in culture flasks at 37 °C according to standard protocols [6]. Virulence was defined as the ability of 5 × 10^5^ trophozoites to produce multiple liver abscesses in hamsters 7 days after intraportal injection. Such virulence was maintained by passing axenic amoebic cultures through hamsters’ livers twice a month, recovering the parasites from 7 days old abscesses and again growing them axenically. Cultures with 72 h expansion were used to perform total lysate of amoebas, cocultures, and in vitro assays.

HepG2 cells from a human-liver-derived cell line were cultured in high glucose DMEM medium supplemented with (10%) fetal bovine serum and (1%) antibiotic cocktail penicillin–streptomycin at 37 °C. HepG2 cells obtained this way were used to perform total cell lysates, cocultures with amoebic trophozoites, and in vitro assays.

### 2.2. Cocultures of Entamoeba histolytica: HepG2

HepG2 cells 3 × 10^6^ were seeded in 10 cm diameter Petri dishes until reaching 60% confluence; subsequently, HepG2 cells were washed with sterile PBS and coincubated with 6 × 10^6^ amoebic trophozoites in 3.5 mL of serum-free TYI medium at 37 °C for 15 min. Elapsed time, cells were recovered with a cell scraper and lysed with lysis buffer (RIPA) supplemented with 10 mM EDTA and 100 mM iodoacetamide.

For microscopic studies, cocultures were performed on sterile coverslips placed in a 12-well multichamber; 6 × 10^4^ HepG2 cells were seeded per well. Subsequently, cell cultures were washed with sterile PBS and coincubated with 6 × 10^4^ (for immunofluorescence staining) or 2 × 10^4^ (for hematoxylin and eosin staining) amoebic trophozoites suspension in 0.5 mL of serum-free TYI medium at 37 °C for 10 or 15 min.

### 2.3. Immunoprecipitation

For each assay, 1 mg of protein, (from coculture) in a final volume of 0.5 mL of lysis buffer supplemented with 10 mM EDTA and 100 mM iodoacetamide was used as the starting sample.

The preclearing of the sample was performed with 60 µL Agarose Protein g (APG) for 2 h at 4 °C under constant stirring; after that time, samples were centrifuged at 12,000 rpm for 5 min at 4 °C, and the supernate was recovered. The supernate (precleared sample) was incubated for 2 h at 4 °C under constant stirring with 10 μL goat anti-c-Met receptor antibody (SIGMA H9786). In parallel, 60 µL APG were incubated with 14 µL of 10% bovine serum albumin (BSA) to prevent unspecific binding, APG-BSA was centrifuged at 3000 rpm, and the supernate was discarded. Subsequently, the precleared sample treated with antibody and BSA–APG were mixed and incubated overnight at 4 °C under constant stirring. Samples were centrifuged at 12,000 rpm for one minute at 4 °C and washed 6 times with 200 μL wash Buffer.

### 2.4. Western Blot

Western blot was performed as described elsewhere [7]. Total cell lysates from cultures, cocultures, or immunoprecipitated samples were resolved by 7.5% SDS–PAGE. Molecular weight markers were precision plus protein dual-color standards, BIO-RAD catalog #161-0374. Proteins were transferred to nitrocellulose membranes. Membranes were blocked with 5% nonfat dry milk in TBS-T for 1 h at room temperature and then incubated overnight at 4 °C with the corresponding antibodies: rabbit anti-Gal lectin (0.4 μg/mL made in our laboratory) [6], goat anti-human c-Met receptor (0.2 μg/mL SIGMA H9786), or goat anti-human HGF (0.2 μg/mL SIGMA H7157). Membranes were washed with TBS-T and incubated with goat anti-rabbit IgG (H + L) (1/60,000 Cell signaling 7074) or bovine anti-goat IgG (H + L) (1:10,000 Santa Cruz Biotechnology sc-2350), all conjugated to horseradish peroxidase for 90 min at room temperature. After washing with TBS-T, antibody-reactive proteins were detected by chemiluminescence.

### 2.5. Hematoxylin and Eosin Staining

Four groups were used in duplicate: (1) HepG2 cells, (2) HepG2 cells + *E. histolytica*, (3) HepG2 cells pretreated with 200 ng/mL HGF (1 h) + *E. histolytica*, and (4) HepG2 + *E. histolytica* pretreated (30 min) with 180 mM Galactose. The incubation with the trophozoites was performed in a 3:1 ratio (HepG2:amoebas) in 0.5 mL of TYI medium at 37 °C for 15 min.

After completion of the different treatments, the coverslips were washed once with PBS, fixed for 30 min in 4% PFA, and postfixed in 0.4% PFA; finally, they were stained with hematoxylin and eosin. Micrographs were taken with Nikon microscope DMX1200 and processed with Nikon ACT-1 software (Version 2.63, Nikon, Tokyo, Japan)

### 2.6. Immunofluorescence Assay

After coculture, the coverslips were washed once with PBS and fixed for 10 min in 4% PFA. Samples were washed thrice with PBS at room temperature and then were incubated in 1% BSA for 1 hr; after that time, samples were incubated whit a dilution (2 μg/mL) of goat anti-human c-Met receptor antibody (SIGMA H9786) overnight at 4 °C. The next day samples were washed three times with PBS and by last were incubated with bovine anti-goat antibody coupled to CF 488A fluorophore (1:1000 SIGMA SAB4600233-2) for 90 min; the preparations were washed thrice with PBS and then incubated with a dilution of rabbit anti-Gal lectin antibody (1 μg/mL, the one made in our laboratory) overnight at 4 °C. The next day samples were washed three times with PBS and then were incubated with goat anti-rabbit antibody coupled to rhodamine fluorophore (1:2000 Jackson Immuno Research 111-295-144) for 90 min. Subsequently, three washes were carried with PBS, and coverslips were mounted on slides with fluorescence mounting medium. The next day sealant was placed around the coverslips. The confocal micrographs were obtained by fluorescence confocal microscopy Olympus FV1000 Upright BX61WI at the National Laboratory for Advanced Microscopy, Biotechnology Institute from UNAM and were processed with “FV Viewer 4.2b software (Copyright © 2003-2016 OLYMPUS CORPORATION, Allentown, PA, USA).

## 3. Results

### 3.1. Expression of Gal Lectin in Amoebic Trophozoites and c-Met in HepG2 Cells

**Figure 1:**
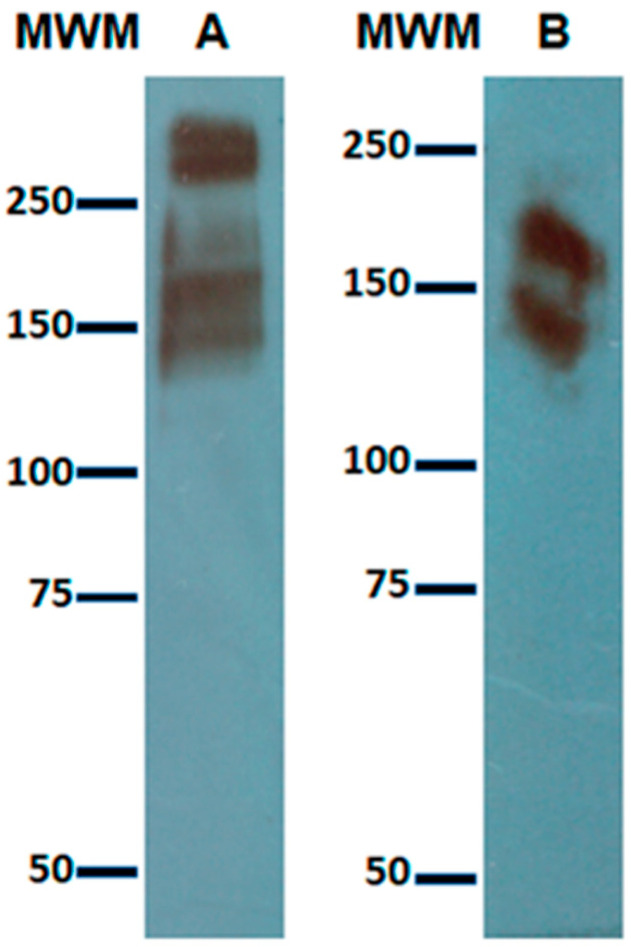
Immunodetection of Gal lectin Expression in trophozoites from *E. histolytica* HM-1:IMSS and of c-Met in HepG2 cells. Total lysates from (**A**) a pellet of amoebic trophozoites or (**B**) a pellet of HepG2 cells were separated by SDS PAGE and subjected to electrotransfer onto nitrocellulose membranes. Immunodetection was performed with (**A**) rabbit polyclonal anti-Gal lectin (prepared at our lab) for amoebic lysate or (**B**) a commercial goat anti-c-Met receptor for HepG2 lysate, respectively. MWM: molecular weight markers (KDa).

Amoebic lectin and c-Met were expressed in trophozoites and in HepG2 cells, respectively. Immunodetection of the Gal lectin in the amoebic lysate was performed using a polyclonal rabbit anti-Gal lectin antibody generated at our lab [7]. We can see three main bands (Figure 1A) with approximate molecular weights of 260 kDa, that matches with the heterodimer referred by W.A. Petri et al. [8] (constituted by the heavy subunit covalently linked to the light one), another band of 170 kDa that matches to the heavy subunit alone and a band with a molecular weight very near to 150 kDa, corresponding to the unglycosylated form of the heavy subunit (143 kDa) or to the intermediate subunit described by Cheng et al. [9], which although not covalently linked to the heterodimer, in many purified samples of the Gal lectin appears as traces [1] maybe because the three subunits function as a complex and are assembled as a whole at the membrane [10].

Immunodetection of the c-Met receptor was performed using a commercial polyclonal antibody (Sigma H9786): two bands are present (Figure 1B)—one with a molecularweight of 150 kDa, which is the molecular weight reported by Giordano et al. [11] for the β subunit, and the other with an approximate molecular weight of 190 kDa that corresponds to the heterodimer constituted by an α chain and a β chain. Thus, we were certain that the c-Met receptor was expressed in our culture of HepG2 cells.

### 3.2. Identification of the Gal Lectin by Anti-HGF Antibody

**Figure 2:**
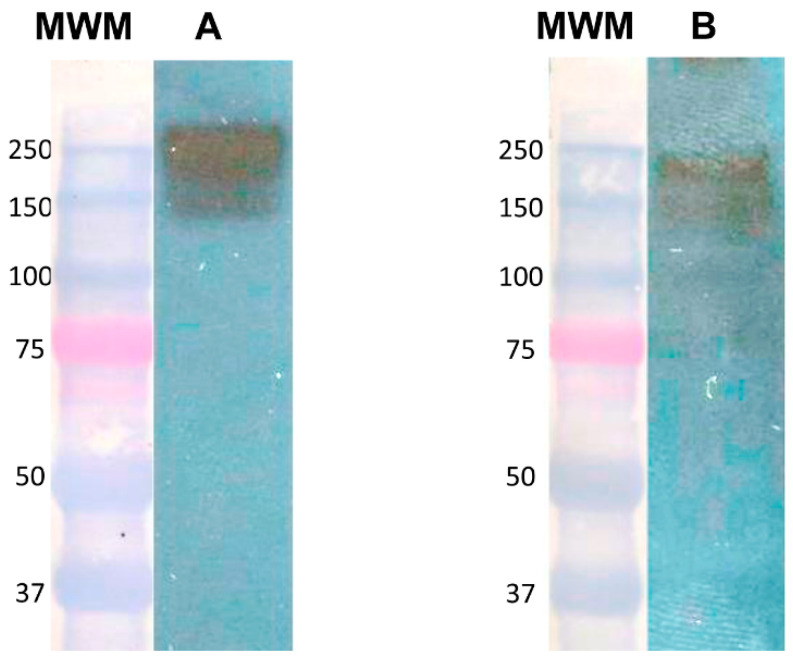
Anti-HGF antibodies identified the heavy subunit from Gal lectin in the total amoebic lysate. A total lysate from *E. histolytica* trophozoites was subjected to Western blot analysis. Immunodetection was performed with anti-Gal lectin antibodies (**A**) or anti-recombinant HGF antibodies (**B**). MWM: molecular weight markers.

Dodson et al. [2] had reported that not only the recombinant CRD but even the purified Gal lectin competed with HGF (the natural ligand) for binding to the c-Met receptor in competition binding assays; therefore, we hypothesized that anti-HGF antibodies could recognize specifically the Gal lectin and/or anti-Gal lectin antibodies could recognize HGF, since these two molecules share a sequence identity. To test this, we performed a Western blot of a total lysate from trophozoites and revealed it with an anti-Gal lectin antibody (Figure 2A) or with a commercial anti-HGF antibody (Figure 2B). The anti-HGF antibodies recognized two bands identical to those recognized by the anti-Gal lectin antibody—one corresponding to the glycosylated heavy subunit with a molecular weight of 170 kDa and another with a smaller molecular weight, which may correspond to the unglycosylated form. The anti-HGF antibody does not recognize the band of 260 kDa that corresponds to the heterodimer of Gal lectin, although it contains the heavy subunit covalently linked to the light one. One explanation for this fact is that the antibody may not have access to the identity region if it is in the heterodimeric form.

In parallel, we transferred recombinant HGF and assayed recognition with an anti-Gal lectin antibody, but it did not recognize HGF (not shown). This result demonstrates that from all proteins in the amoebic lysate the heavy subunit of Gal lectin is the only one that shares an identity sequence with human recombinant HGF.

### 3.3. Immunoprecipitation: Interaction of c-Met and Gal Lectin

**Figure 3:**
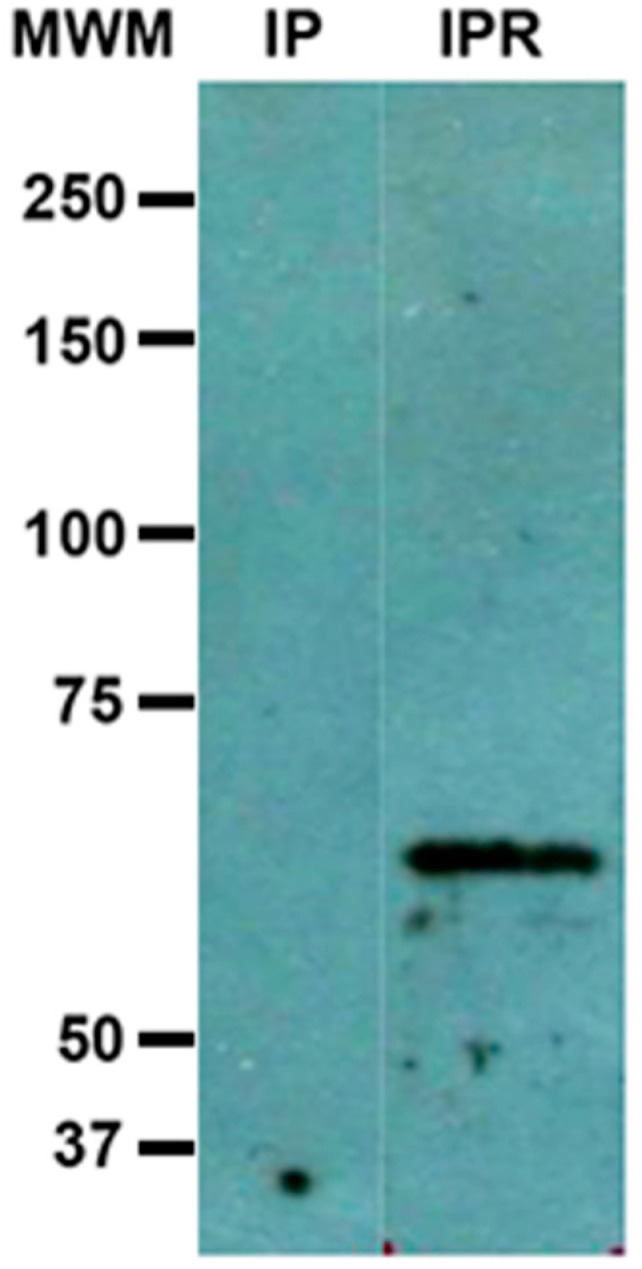
Identification of Gal Lectin in anti-c-Met receptor immunoprecipitate from an *E. histolytica*:HepG2 coculture. The c-Met immunoprecipitate from an *E. histolytica*:HepG2 coculture cell extract was separated by SDS–PAGE and electrotransferred onto nitrocellulose membrane, immunodetection was performed with the rabbit polyclonal anti-Gal lectin antibody. IP: immunoprecipitate not reduced; IPR: immunoprecipitate under reduced conditions with 2-β mercaptoethanol. MWM: molecular weight markers.

The immunoprecipitated sample was subjected to Western blot analysis. When revealed with anti-Gal lectin antibodies, there was no band detected in the immunoprecipitate; however, when the sample was reduced with 2-β mercaptoethanol, a well-defined band with an approximate molecular weight of 60 kDa was revealed (Figure 3). We expected to detect the heavy subunit in any of its forms—as a heterodimer (260 kDa) or as the heavy subunit (170 kDa)—but this was not observed. This peptide with an approximate molecular weight of 60 kDa was recognized by anti-Gal lectin antibodies; it probably came from Gal lectin. It seems as if antibodies had no access to epitopes until disulfide bonds are reduced. Sometimes, in our lab, when treating pure samples of the amoebic Gal lectin with a reducing agent such as β-mercaptoethanol or dithiothreitol (DTT), bands of 260 and 170 kDa vanished, and instead, a band with a molecular weight around 60 KDa appears and is recognized by our anti-Gal lectin antibody, so we think it is part of the Gal lectin.

### 3.4. Optical Microscopy of the E. histolytica Trophozoites: HepG2 Cocultures

**Figure 4:**
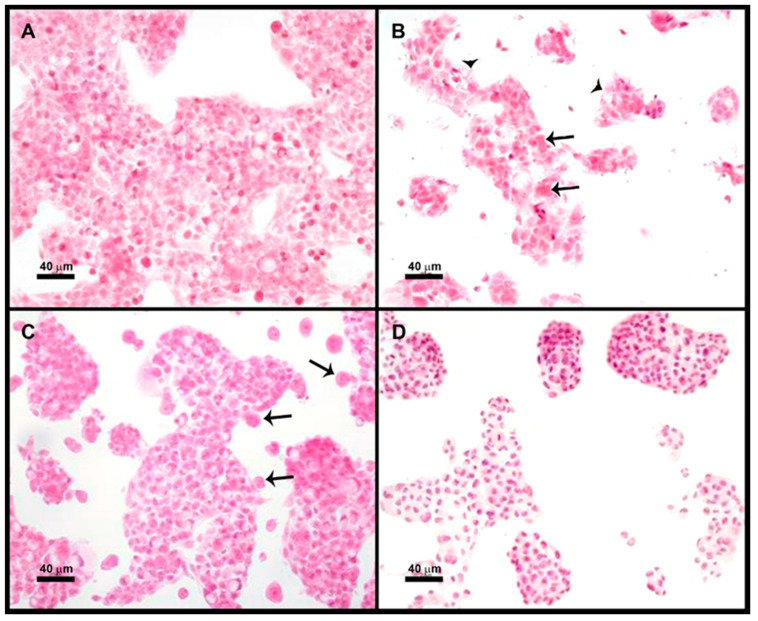
Prevention of cytotoxic effect caused by amoebic trophozoites to HepG2 cells by HGF or galactose pretreatment: (**A**) HepG2 cells, as shown on hematoxylin–eosin stained slides without any processing; (**B**) HepG2 cells incubated with *E. histolytica* (arrows indicate amoebas, arrowheads indicate cell damage); (**C**) HepG2 cells pretreated with HGF 200 ng/mL (1 h) and *E. histolytica;* note that amoebas (arrows) adhere to cells, but no cell damage is observed; (**D**) HepG2 incubated with *E. histolytica* pretreated with galactose 180 mM (30 min); note that there are no amoebas adhered and no cell damage.

We performed a microscopic study of amoebic trophozoites coincubated with HepG2 cells for 15 min in a 3:1 ratio. Amoebic trophozoites caused a cytotoxic effect on HepG2 cells (Figure 4B). The cytotoxicity induced by trophozoites, but not their adherence to cells, can be prevented by pretreatment of cells with 200 ng/mL HGF; arrows indicate adhered trophozoites (Figure 4C). Treatment of amoebas with 180 mM galactose for 30 min prior to their coincubation with HepG2 cells prevents both adherence and cytotoxicity of amoebic trophozoites (Figure 4D), as previously described for Madin–Darby canine kidney (MDCK) cells [12].

### 3.5. Fluorescence Confocal Microscopy Study: Colocalization of Gal Lectin and c-Met Receptor

**Figure 5:**
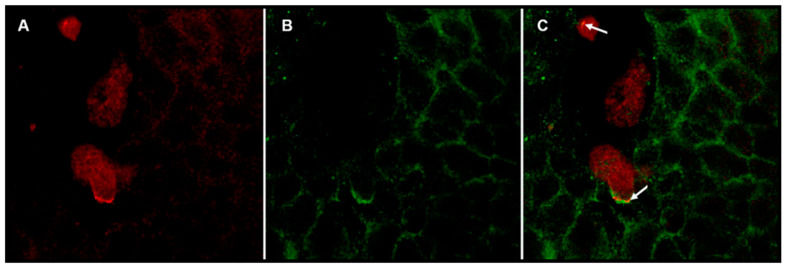
Interaction between *E. histolytica* Gal lectin and HepG2 c-Met receptor. Fluorescence confocal microscopy of Amoebas:HepG2 coculture. Amoebic trophozoites and HepG2 cells were cocultured for 10 min in a 1:1 ratio, cells were then fixed with 4% paraformaldehyde and stained with rabbit polyclonal anti-Gal lectin antibody in red (**A**) and anti-c-Met receptor antibody in green (**B**). Merge (**C**) arrows show contact area between both cell types, evidencing interaction of Gal lectin from *E. histolytica* trophozoites with c-Met receptor expressed in HepG2 cells.

We used another approach to determine if Gal lectin interacts with c-Met in a coculture assay; in this method, we coincubated *E. histolytica* trophozoites with HepG2 cells in a 1:1 ratio for 10 min, and fixed preparations were treated with our homemade rabbit polyclonal anti-Gal lectin antibody and anti-c-Met receptor antibody, then with a secondary that fluoresces in red (Figure 5A) or in green (Figure 5B), respectively. The merge of the two images indicated some yellow perimeter, which enabled us to suggest the interaction of this pair of molecules at sites of contact (Figure 5C).

## 4. Discussion

*Entamoeba histolytica* has a potent cytotoxic activity on diverse cell types. Even though the mechanism by which the parasite induces killing has not been completely elucidated, one thing is for certain: adherence of the parasite is required, and adherence is achieved by the Gal lectin, as the presence of an excess of galactose prevents adherence and target killing [12,13]. In addition to mediating adherence, Gal lectin may also participate in the killing. Maybe the best proof that such is the case resulted from the fact that a monoclonal anti-Gal lectin antibody blocked cytotoxicity without blocking adherence [14]. On the other hand, the reported identity of the sequence of the heavy subunit of Gal lectin with the HGF [2], which could be fortuitous, was suggestive for a parasite with a special tropism for the liver. The aim of this work was to analyze the possible interaction of the amoebic trophozoite’s Gal lectin with the c-Met receptor in the surface of cells derived from the human liver.

The recognition of the heavy subunit of the Gal lectin by the anti-HGF antibodies from all the proteins present in the lysate of a pellet from *E. histolytica* trophozoites indicated that although the reported sequence identity was slight, it was enough to be recognized. The fact that our anti-Gal lectin did not recognize recombinant HGF may be explained because these antibodies are polyclonal and were not purified with an affinity method as were those anti-HGF antibodies.

Results from immunoprecipitation with anti-c-Met antibodies revealed under re-duced conditions a 60 kDa peptide identified by anti-Gal lectin antibodies, which may be actually part of the Gal lectin. We do not consider that this result is a cross-reaction of any of the antibodies used in the test. As we mentioned above in Section 3.3, it has happened to us, when working with pure samples of Gal lectin (obtained as described in [7]), that the band of 260 kDa, originally present and recognized by anti-Gal antibody, is no longer apparent when the sample is treated with a reducing agent, but a new 60 kDa band ap-pears that is recognized by the same antibody. In those cases, the initial sample comes ex-clusively from a total amoebic lysate, and therefore cannot be attributed to a cross-recognition of a protein from another cell type, for example from HepG2 cells. how-ever, it would be necessary to analyze our “pure” sample by means of two-dimensional electrophoresis, to have a more complete image of the sample components, we are currently working on it. A plausible explanation to this result would be once bound to its ligand (probably c-Met) the action of the rhomboid protease [15] on the Gal lectin generates a peptide with a smaller molecular weight.

As previously mentioned, it was difficult to discern if the Gal lectin participates directly in the cytotoxicity of the parasite or is the first step in target cell killing. We assayed a microscopic study of cocultures of trophozoites + HepG2 cells; as expected, both adherence and cytotoxicity of the parasite on HepG2 cells can be prevented by pretreatment of amoebas with galactose; however, pretreatment of HepG2 cells with HGF inhibited cytotoxicity but not adherence of amoebas. These results seem to indicate that amoebas need contact c-Met for cytotoxicity. Therefore, amoebic Gal lectin could be participating in adherence and cytotoxicity.

Finally, by means of confocal fluorescence microscopy, we demonstrated that in cocultures of *E. histolytica* trophozoites and HepG2, Gal lectin colocalizes with c-Met, supporting the idea that this pair of molecules interact with each other.
